# Structurally Enhanced Colloidal Molecules via Carbene‐Mediated Covalent C─H Insertion Crosslinking

**DOI:** 10.1002/advs.74940

**Published:** 2026-03-23

**Authors:** Liwei Dai, Chongyang Yao, Xiancheng Zhang, Weijia Kong, Mingxing Liu, Huibin He, Yutao Sang, Zhihong Nie

**Affiliations:** ^1^ State Key Laboratory of Molecular Engineering of Polymers Department of Macromolecular Science Fudan University Shanghai People's Republic of China

**Keywords:** colloidal molecules, photo‐crosslinking, polymer ligands, self‐assembly of nanoparticles, structural stability

## Abstract

Achieving high structural stability of colloidal molecules (CMs) is crucial for their implementation in constructing functional materials and devices, but remains challenging. Here, we develop a universal strategy based on nonspecific covalent crosslinking of surface ligands for markedly enhancing the stability of CMs while fully preserving their structural integrity and physicochemical properties. The resulting crosslinked CMs exhibit exceptional resistance to alkaline conditions (up to pH = 12), high salt concentrations (150 mM), external electric fields, and hydrogen‐bonding perturbations. These achievements stem from the rational design of the crosslinker *3G*, which involves shielding aliphatic C–H bonds, extending the spacing between reactive sites, and increasing the number of active sites, thereby enabling efficient bridging of adjacent particles within CMs under mild reaction conditions. The nonspecific nature of this *3G*‐based approach affords broad applicability, setting it apart from conventional stabilization methods that require purpose‐designed crosslinkable ligands. This stabilization approach can be readily extended to CMs with complex three‐dimensional architectures, expanding opportunities for exploiting diverse CM libraries across a wide range of applications.

## Graphical abstract

This work presents a nonspecific covalent crosslinking strategy to significantly enhance the structural stability of colloidal molecules (CMs) without compromising their inherent structural and physicochemical properties by rationally designing a diazo‐based crosslinker. In contrast to uncrosslinked CMs, the crosslinked ones exhibit exceptional resistance to alkaline conditions, high salt concentrations, external electric fields, and hydrogen‐bonding perturbations.

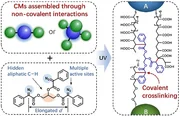

## 1 Introduction

Inorganic nanoparticles (NPs), owing to quantum confinement effects, exhibit intriguing optical, electrical, thermal, and magnetic properties that surpass those of their bulk counterparts [1, 2]. Assembling NPs into small, well‐defined clusters, particularly colloidal molecules (CMs), can further enhance these intrinsic properties and/or generate new characteristics arising from interactions among the plasmons, excitons, or magnetic moments of the constituent particles [3, 4]. Such collective effects hold promise for applications in catalysis [5], sensing [6], energy harvesting [7], and optoelectronic devices [8]. Owing to their precise and predictable architectures, CMs can mimic the structural characteristics of real molecules, and their size advantage over conventional molecules makes them visually accessible analogues for studying diverse molecular phenomena (e.g., crystallization, melting, and glass transitions) that are otherwise difficult to observe directly at the molecular scale [9, 10]. In addition, CMs are attractive building blocks for creating hierarchically organized materials because they feature multiple, tunable interactions. It is noteworthy that the collective properties, molecule‐like behaviors, and hierarchical assembly of CMs critically depend on their structural stability [11, 12]. Therefore, controlling interparticle interactions to obtain structurally robust CMs is essential for their practical applications.

CMs formed through noncovalent interactions such as electrostatic forces, hydrogen bonding, hydrophobic interactions, halogen bonding, and dipole–dipole interactions are typically structurally unstable under external chemical or physical stimuli. For instance, electrostatically assembled CMs are extremely sensitive to changes in ionic strength and pH [13], while hydrophobically driven assemblies tend to disassemble in response to minor variations in solvent polarity [14]. For assemblies stabilized by hydrogen bonding, the introduction of additional donors or acceptors often perturbs interparticle interactions, ultimately leading to structural disintegration [15]. To mitigate these instabilities, various stabilization strategies have been developed, including polymer or silica encapsulation [16, 17], UV irradiation or thermal fusion [17, 18], and welding by lasers [19] or small molecules [20]. However, these approaches often obscure the intrinsic properties of the assemblies, diminish their spatial adaptability, or induce structural distortions. Recently, covalent bonding has been employed to strengthen interparticle interactions in NP assemblies, which typically involves the deliberate design of reactive ligands to replace native NP ligands, followed by covalent bonding‐driven assembly of NPs [21, 22, 23]. Such methods, however, are constrained by the laborious synthesis of specialized ligands and the narrow range of compatible NP–ligand combinations. Therefore, there is an urgent need for a simple and efficient strategy to construct structurally robust CMs without compromising their original architectures and properties.

**FIGURE 1:**
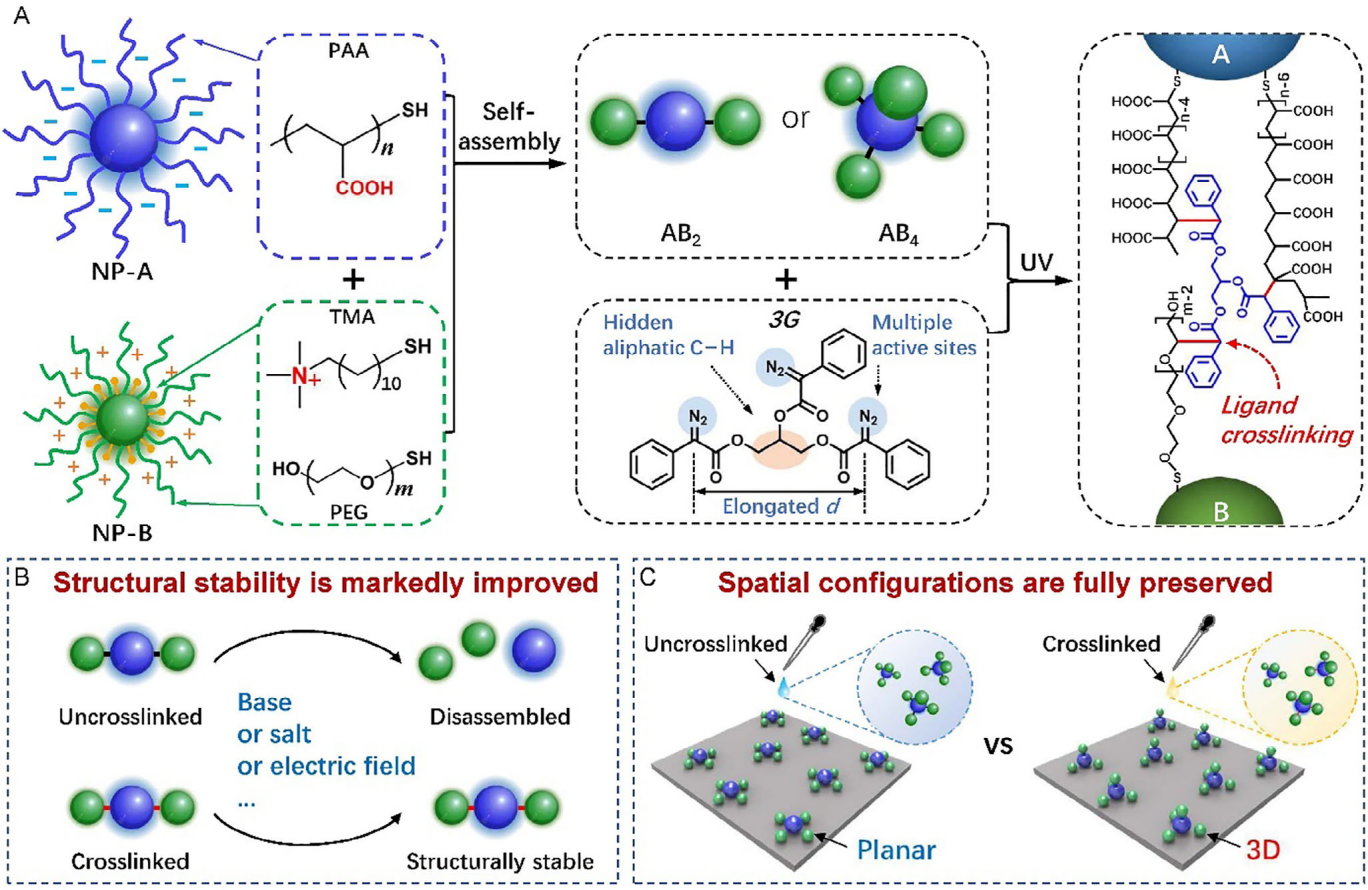
(A) Schematic illustration of the strategy for stabilizing CMs, including electrostatic assembly of NPs into defined geometries followed by nonspecific covalent crosslinking between diazo compounds and surface ligands. (B) Stability of AB_2_‐type CMs under various harsh conditions. (C) Comparison of the structural stability of 3D AB_4_‐type CMs in the dry state before and after covalent crosslinking.

Herein, we present a universal strategy that significantly enhances the structural stability of CMs across diverse spatial configurations through nonspecific covalent crosslinking of native surface ligands, without the need for specially designed crosslinkable ligands. As a representative model, noncovalent CMs assembled from two types of complementary polymer‐grafted NPs via synergistic electrostatic and hydrogen bonding interactions were selected for covalent crosslinking (Figure 1A, left). The surface ligands capped on the center particles (polyacrylic acid, PAA) and coordinating particles (poly(ethylene glycol), PEG, and (11‐mercaptoundecyl)‐*N,N,N*‐trimethylammonium bromide, TMA) contain rich aliphatic C─H bonds, providing abundant reactive sites for subsequent nonspecific C─H insertion reactions. Three‐armed diazo‐based crosslinkers (*3G*) were employed as additives. Through iterative optimization over three generations—including the shielding of aliphatic C─H bonds, extension of the distance between active sites, and an increase in the number of active sites—the *3G* exhibits a higher molar extinction coefficient (*ɛ*) and superior crosslinking efficiency compared with its first‐ and second‐generation counterparts (abbreviated as *1* and *2G*). Upon UV irradiation, *3G* generates highly reactive carbenes that undergo C─H insertion with the surface ligands of CMs, forming robust covalent bonds (Figure 1A, right). The *3G*‐based crosslinking effectively stabilizes CMs while largely preserving their intrinsic structural and physicochemical characteristics, which is difficult to achieve with previous approaches [16, 17, 18, 19, 20]. Moreover, the nonspecific nature of the *3G*‐based approach confers broad applicability, distinguishing it from conventional methods [21, 22, 23] that rely on specifically designed crosslinkable ligands. The crosslinked CMs retain their structural integrity under various harsh conditions, including strong alkaline environments (pH 12), high salt concentrations (150 mM), applied electric fields, and disturbances from competitive hydrogen‐bonding species (Figure 1B). In contrast, uncrosslinked CMs readily disassemble even under mild alkaline (pH 10.5) or low‐salt conditions (10 mM). Notably, this strategy enables CMs with high coordination numbers to maintain their three‐dimensional (3D) architecture in the dry state (Figure 1C), opening avenues for evaporation‐ or crystallization‐induced hierarchical assembly of CMs into functional materials.

## 2 Results and Discussion

Diazo compounds constitute a versatile class of small‐molecule crosslinkers distinguished by their mild activation conditions, high crosslinking efficiencies, favorable photochemical behavior, and ease of synthesis relative to traditional crosslinkers (e.g., azides and diazirines) [24, 25, 26]. Motivated by these attributes, we employed diazo‐based crosslinkers to covalently stabilize CMs. Ethyl phenyldiazoacetate (*1G*) serves as a prototypical carbene‐generating crosslinker containing two photodegradable diazo groups. However, the close spatial proximity of these groups predisposes *1G* to self‐coupling side reactions [27], potentially compromising its crosslinking performance. To overcome this limitation, we rationally redesigned the molecular structure of *1G* by hiding aliphatic C─H bonds, extending the distance between active sites, and increasing the number of active diazo moieties (Figure S1A–C). These efforts yielded second‐ and third‐generation analogues, *2* and *3G*, respectively. ^1^H nuclear magnetic resonance (NMR) spectra confirm the successful synthesis of all three crosslinkers (Figures S2–S4), and the detailed synthetic routes are provided in Figures S5–S7. All compounds exhibit strong absorption in the UV region (Figure S8A), with *ɛ* (at 254 nm) of 1.6 × 10^4^, 2.7 × 10^4^, and 4.2 × 10^4^ cm^−1^ M^−1^ for *1G*, *2G*, and *3G*, respectively. Consistent with its higher *ɛ*, *3G* undergoes photolysis at a markedly accelerated rate relative to *1* and *2G* (Figure S8B). Moreover, the multi‐armed branched architecture of *3G* promotes effective bridging between ligands on adjacent particles rather than intraparticle linking. Given its superior structural and photochemical properties, *3G* was selected for the subsequent experiments.

**FIGURE 2:**
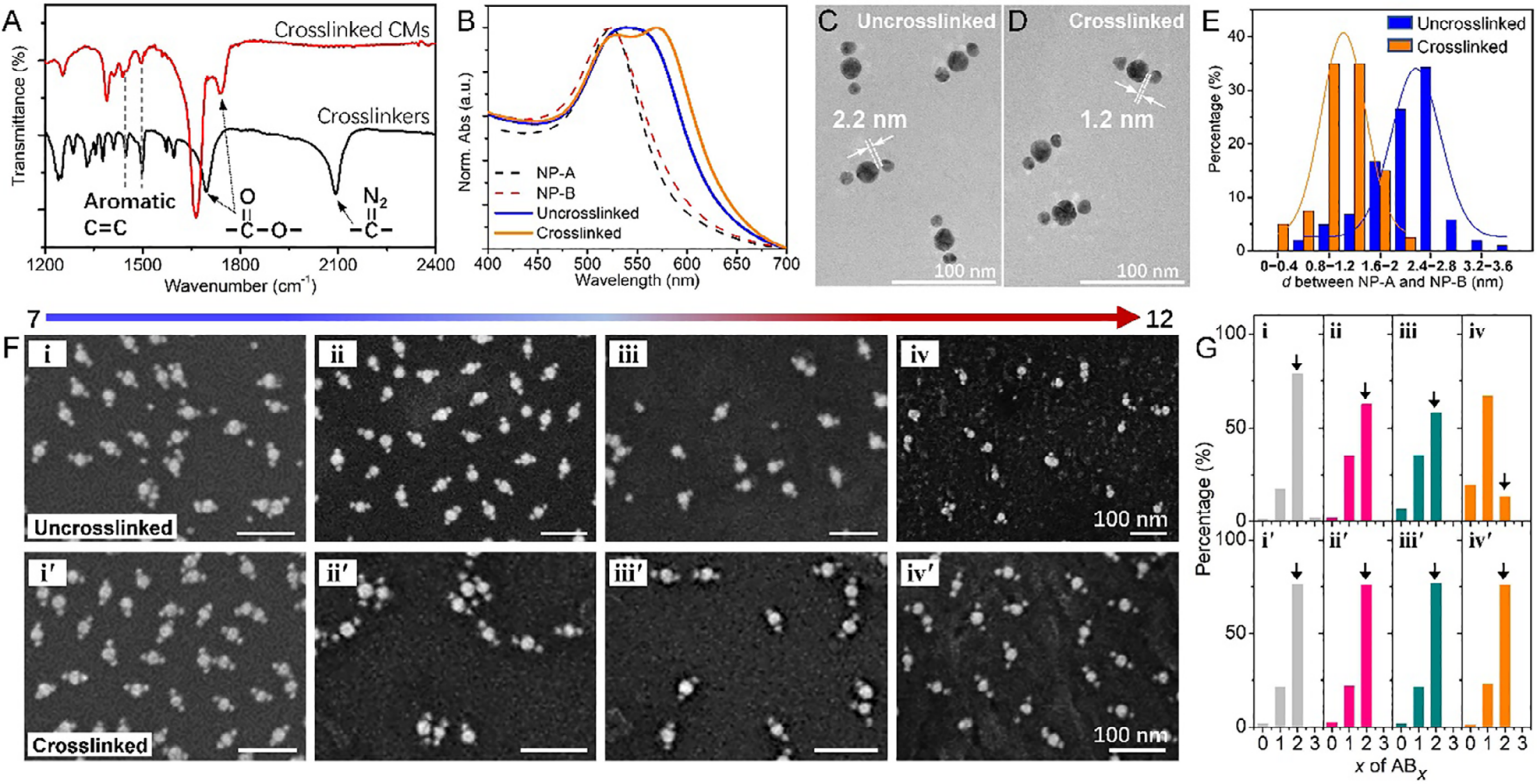
(A) FTIR spectra of the crosslinkers and the resulting crosslinked CMs. (B) UV−vis absorption spectra of NP‐A, NP‐B, AB_2_‐type CMs before and after crosslinking. (C−D) Cryo‐TEM images of AB_2_‐type CMs before and after crosslinking. (E) Corresponding statistical distributions of the interparticle spacing within the CMs. (F) SEM images showing structural evolution of AB_2_‐type CMs as the pH increases sequentially from 7.0 to 10.5, 11.5, and 12.0. (G) Corresponding statistical analysis of AB_x_‐type CM populations as a function of pH.

We employed planar AB_2_‐type and 3D AB_4_‐type CMs, constructed via synergistic electrostatic/hydrogen‐bonding interactions among polymer‐grafted NPs [13], as model systems for illustration. Au NPs of varying diameters (15, 20, and 25 nm; Figure S9A–F) were synthesized and functionalized with complementary polymers to generate four types of building blocks: NP‐A, NP‐A′, NP‐B, and NP‐B′. Specifically, NP‐A comprises 20 nm Au NPs functionalized with PAA (MW = 13.5 kDa), whereas NP‐B consists of 15 nm Au NPs modified with PEG (MW = 20 kDa) and TMA. These two species self‐assemble into AB_2_‐type CMs. NP‐A′ is 25 nm Au NPs grafted with PAA, and NP‐B′ is 15 nm Au NPs modified with PEG (MW = 10 kDa) and TMA, and they form AB_4_‐type CMs. Detailed protocols for CM preparation are provided in the experimental section. *3G* was then added into the systems, and it undergoes photolysis upon irradiation with 254 nm UV light to generate highly reactive carbene species that insert nonspecifically into C─H bonds of surface ligands, thereby establishing covalent crosslinks within AB_2_‐type CMs. Fourier‐transform infrared (FTIR) spectroscopy confirms the occurrence of crosslinking (Figure 2A): the diazo stretching vibration band (C = N_2_) of *3G* at 2091 cm^−1^ disappears after UV irradiation, indicative of complete photodecomposition. Concurrently, the C═O stretching vibration peak of the ester group in *3G* shifts from 1697 to 1746 cm^−1^, suggesting the formation of new C─C covalent bonds between *3G* and the surface ligands. Aromatic C═C bands originating from the phenyl ring of *3G* remain evident in the FTIR spectrum of the crosslinked CMs, further corroborating covalent linkage. The UV–vis absorption spectrum of the crosslinked CMs shows a distinct redshift and emergence of a new absorption peak around 570 nm relative to that of the uncrosslinked CMs (Figure 2B). The original peak at 528 nm is attributed to the transverse plasmon coupling mode, while the new peak at 570 nm corresponds to the longitudinal plasmon coupling mode along the interparticle axis and is sensitive to changes in interparticle spacing [28]. The appearance of the longitudinal mode suggests a reduction in interparticle spacing following covalent locking. To eliminate potential artifacts associated with capillary forces arising from solvent evaporation during sample preparation, the CMs were rapidly vitrified in liquid nitrogen to preserve their solution‐state configurations, followed by imaging via cryogenic transmission electron microscopy (cryo‐TEM). Both Cryo‐TEM images and statistical analyses (Figure 2C–E) reveal that the average interparticle spacing within the CMs decreases from 2.2 ± 0.5 to 1.2 ± 0.4 nm after crosslinking.

We compared the structural stability of uncrosslinked and crosslinked AB_2_‐type CMs under different harsh conditions, including strong alkaline environments, high salt concentrations, external electric fields, and disturbances from competitive hydrogen‐bonding species. As shown in Figure 2F‐i–iv, the uncrosslinked AB_2_‐type CMs progressively disassemble into AB‐type structures and free individual NP‐A as pH increases from the initial value of 7 to 12. Quantitative analysis (Figure 2G, top) shows that the proportion of AB_2_‐type structures decreases from 78.9% to 13.4% as pH increases, while the proportions of AB and NP‐A species increase from 17.6% and 1.5% to 67.2% and 19.4%, respectively. To understand the disassembly behavior of uncrosslinked CMs under alkaline conditions, we monitored the changes in hydrodynamic diameter (*D*_h_) and zeta potential of the constituent building blocks, NP‐A and NP‐B, across the same pH range. With increasing pH, the *D*_h_ of NP‐B remains almost unchanged, whereas that of NP‐A increases substantially from 22 to approximately 43 nm (Figure S10), indicating enhanced steric repulsion between NP‐A and NP‐B. Meanwhile, as the medium changes from neutral to alkaline, the negative surface charge of NP‐A slightly decreases, while NP‐B transitions from positively charged to nearly neutral (Figure S11), leading to a reduction in electrostatic attraction between the two components. Consequently, the combined effect of enhanced steric repulsion and weakened electrostatic attraction drives the disassembly of the uncrosslinked CMs under alkaline conditions. In contrast, the crosslinked AB_2_‐type CMs, stabilized by robust covalent bonds between particles, retain structural integrity throughout the same pH range (Figure 2F‐i′–iv′), with the proportion of AB_2_ population remaining nearly constant (Figure 2G, bottom). The crosslinker amount‐dependent experiments further demonstrate that achieving such robust stabilization requires a minimum of approximately 3 µmol of *3G* (Figure S12). Furthermore, under identical reaction conditions (e.g., with the same amount of crosslinker added), the CMs crosslinked with *3G* exhibit superior structural stability compared with those crosslinked with *1G* or *2G* (Figure S13). This enhanced robustness indicates the better crosslinking performance of *3G*, which can be attributed to the rational design of its molecular structures.

**FIGURE 3:**
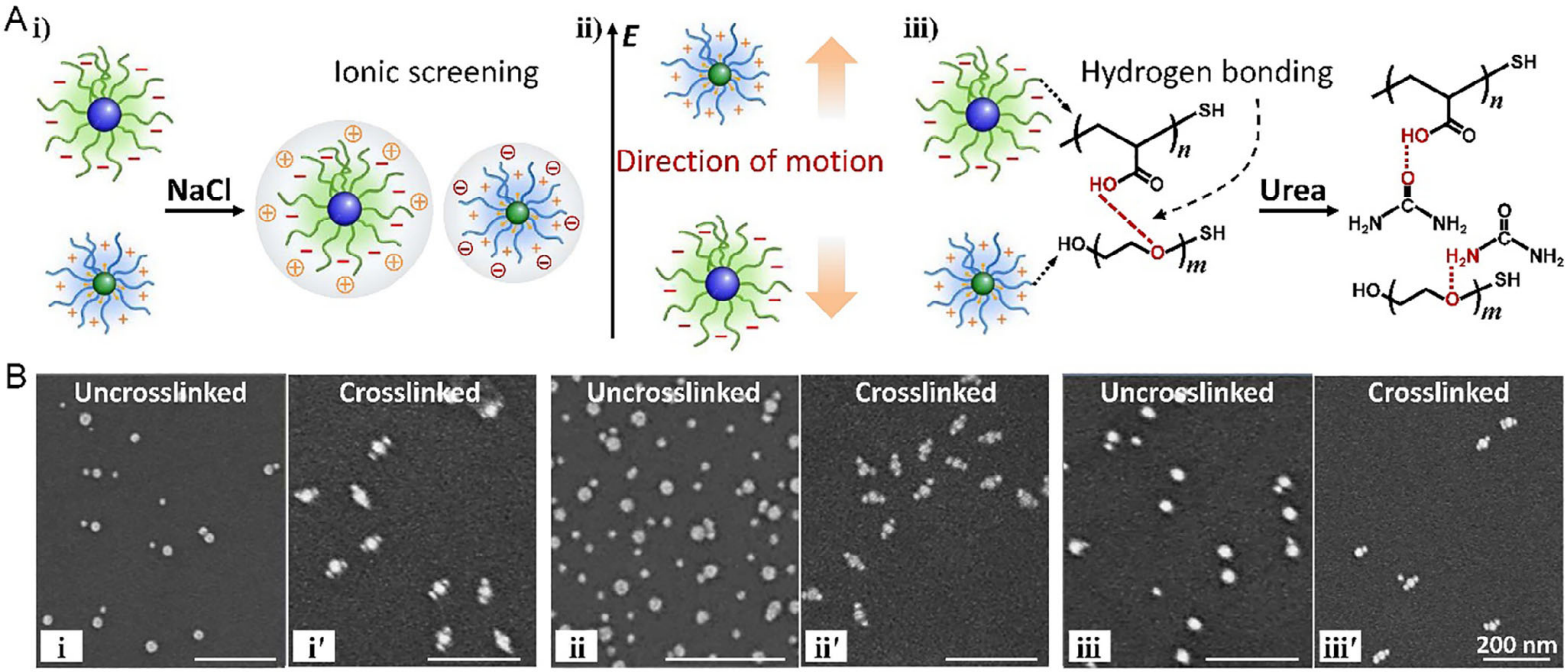
(A) Stability tests of AB_2_‐type CMs before and after covalent crosslinking under various harsh conditions, including: (i) high salt concentrations (150 mM), (ii) external electric fields, and (iii) disturbances from competitive hydrogen‐bonding species. (B) Corresponding SEM images of the CMs under the respective test conditions.

We further evaluated CM stability under high salt concentrations. When dispersed in ionic media, electrostatically stabilized CMs suffer from weakened interparticle attraction due to ionic screening [29, 30] (Figure 3A‐i). As expected, the uncrosslinked AB_2_‐type CMs dissociate into AB and NP‐A species in a 150 mM NaCl solution (Figure 3B‐i). Even when the NaCl concentration is reduced by an order of magnitude (e.g., 10 mM), the uncrosslinked assemblies remain unstable and fail to retain their original configuration (Figure S14). By contrast, the crosslinked AB_2_‐type CMs maintain their structural integrity in a 150 mM NaCl solution (Figure 3B‐i′), owing to the robust covalent linking between the constituent particles. Additionally, purification strategies involving electric fields, such as electrophoresis, are highly effective for improving the purity of CMs [31, 32], but their successful implementation relies critically on the structural stability of the CMs under applied fields. Experimental observations show that the uncrosslinked AB_2_‐type CMs disassemble in an electric field (Figure 3B‐ii), as NP‐A and NP‐B—bearing opposite charges—migrate in opposite directions (Figure 3A‐ii). Under identical conditions, the covalently crosslinked CMs retain their AB_2_‐type configuration (Figure 3B‐ii′). In this system, hydrogen bonding provides an additional stabilizing force alongside electrostatic attraction, yet such interactions are readily disrupted by competitive hydrogen‐bonding species such as urea (Figure 3A‐iii), leading to rapid structural disintegration of the uncrosslinked CMs (Figure 3B‐iii). Conversely, the covalently crosslinked AB_2_‐type CMs remain intact under the same conditions (Figure 3B‐iii′).

**FIGURE 4:**
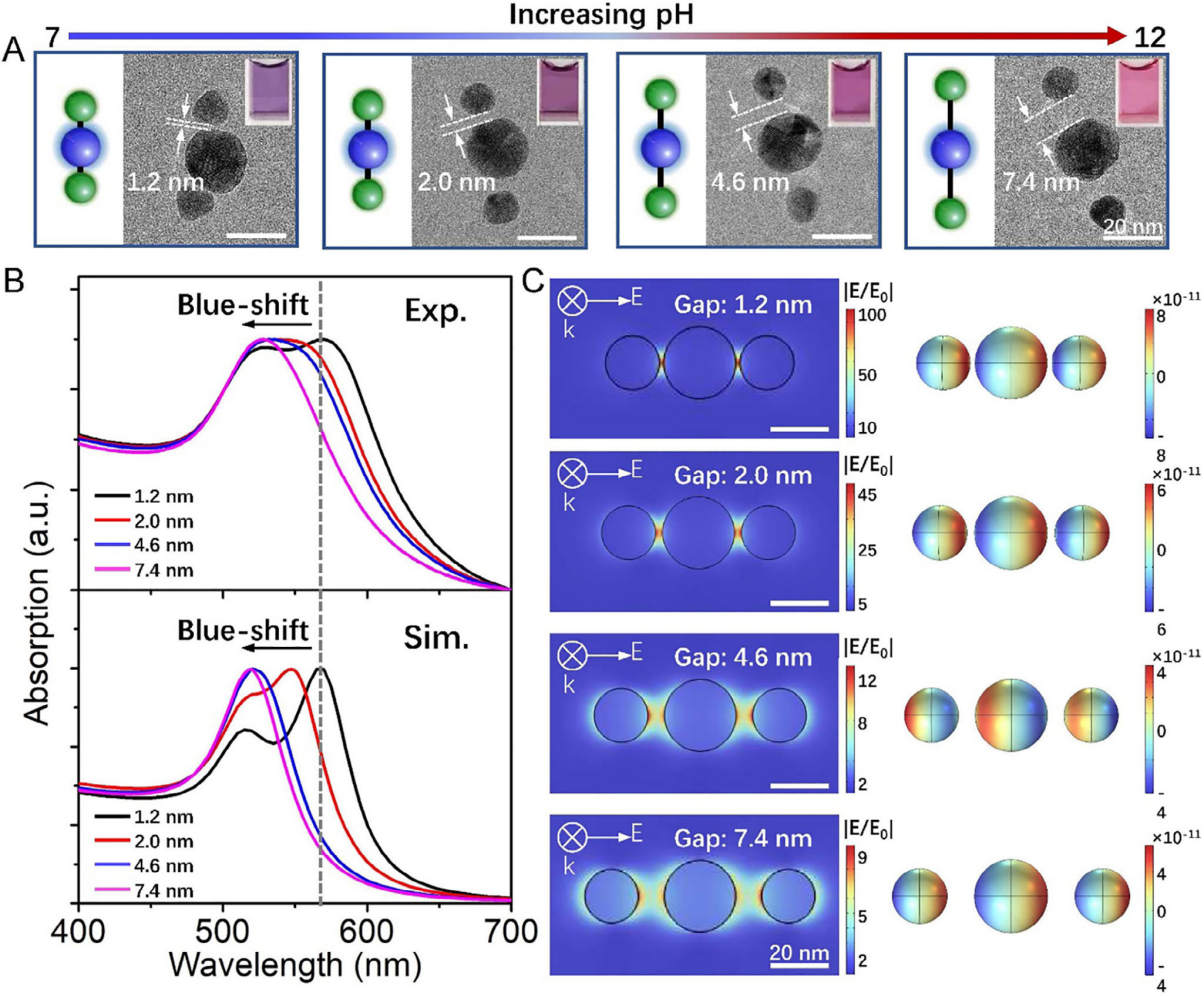
(A) Cryo‐TEM images and corresponding photographic insets of crosslinked AB_2_‐type CMs, revealing pH‐triggered changes in bond length. The pH increases sequentially from 7.0 to 10.5, 11.5, and 12.0. (B) Experimental and simulated UV–vis absorption spectra of the CMs as a function of the bond length. (C) Corresponding near‐field electric field distributions and simulated surface charge densities at the central wavelengths of the dominant dipole–dipole bonding modes.

Importantly, *3G* induces covalent crosslinking between polymer ligands on the NP surface rather than directly welding or fusing the NPs. Thus, the crosslinked CMs combine high structural stability and retained flexibility, yielding molecule‐like behavior that is difficult to achieve with previous stabilization strategies [16, 17, 18, 19, 20]. For example, the crosslinked AB_2_‐type CMs exhibit reversible modulation of interparticle “bond” length (Figure 4A). Cryo‐TEM images reveal that the average bond length of the crosslinked CMs expands from 1.2 ± 0.4 to 7.4 ± 2.8 nm with increasing pH, accompanied by a visible color change in the dispersion from purple to red (Figure 4A, insets). This bond elongation stems from the pH responsiveness of PAA grafted onto the NP‐A surfaces: As pH increases, the protonation degree of carboxyl groups in PAA decreases, promoting chain extension and thereby increasing the interparticle spacing (i.e., the bond length). Notably, restoring the solution to pH 7 contracts the bond length to near its original value (Figure S15). The crosslinked CMs also display bond‐length‐dependent plasmonic absorption features (Figure 4B, top). With increasing bond length, the reduced plasmonic coupling between adjacent NPs results in a pronounced blue shift of the longitudinal plasmon coupling band, while the transverse plasmon coupling band remains essentially unchanged. These plasmonic coupling behaviors are even more clearly manifested in the simulated spectra (Figure 4B, bottom). COMSOL modeling reveals that, at a bond length of 1.2 nm, the strong near‐field plasmon coupling between adjacent particles highly concentrates the electric field within the bond region, with a maximum field intensity (|*E*|_max_) reaching approximately 100 times that of the incident field (Figure 4C, left). As the bond length increases from 1.2 to 7.4 nm, the electric field intensity within the gap decays exponentially (Figure S16). In contrast, the corresponding surface charge distributions remain largely unaffected by bond‐length variation, consistently exhibiting a characteristic dipole–dipole‐like pattern (Figure 4C, right).

**FIGURE 5:**
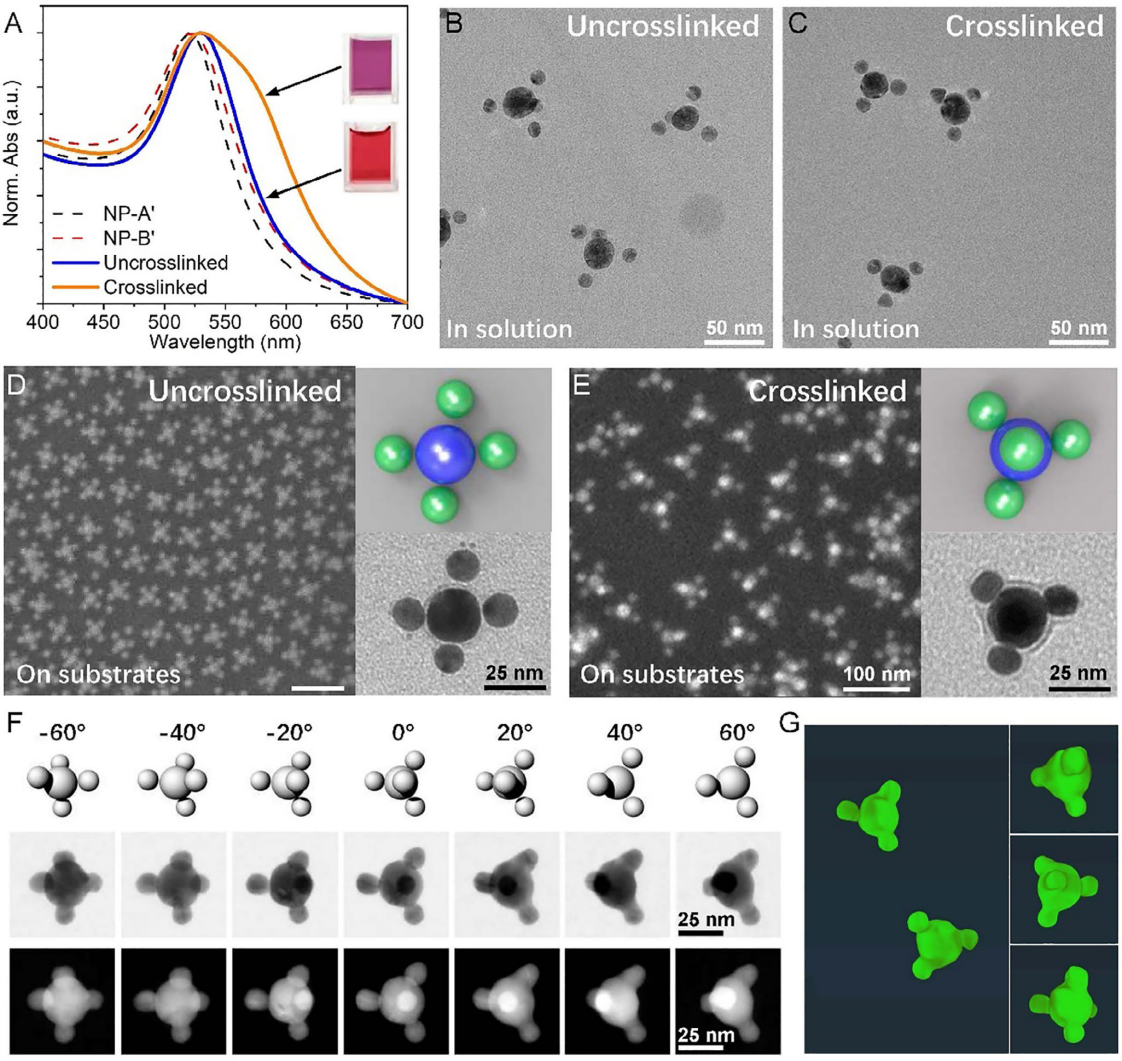
(A) UV−vis absorption spectra of NP‐A′, NP‐B′, AB_4_‐type CMs before and after crosslinking. (B−C) Cryo‐TEM and (D−E) SEM images of AB_4_‐type CMs before and after crosslinking, respectively. (F) Schematic illustration (top), TEM images (middle), and STEM‐HAADF images (bottom) of a crosslinked AB_4_‐type CMs, captured at successive 10° rotations about the *Y*‐axis. (G) 3D reconstruction of crosslinked AB_4_‐type CMs (left), with multiple viewing angles of a single 3D model (right). The 3D model was generated primarily from TEM images acquired at 1° intervals over a *Y*‐axis tilt range of ±60°.

The ligand‐crosslinking‐based stabilization strategy is equally applicable to 3D CMs with higher coordination numbers. Taking AB_4_‐type CMs as an example, crosslinking induces a new absorption peak at approximately 573 nm in the UV–vis spectrum (Figure 5A), accompanied by a reduction in bond length (Figure 5B,C). Interestingly, the uncrosslinked AB_4_‐type CMs with tetrahedral‐like configurations remain stable only in dispersion, as evidenced by cryo‐TEM imaging (Figure 5B). Upon deposition and drying on a substrate, they collapse into planar structures (Figure 5D; Figure S17), likely due to the flexibility of the polymer chains bridging adjacent particles. By contrast, the crosslinked AB_4_‐type CMs preserve their 3D architecture even after drying (Figure 5E), a result of the robust covalent interparticle linkages that stabilize their structural integrity. To further investigate their 3D morphology, we conducted scanning transmission electron microscopy (STEM) with sample tilting in 10° increments around the *Y*‐axis. The resulting angle‐dependent projections align well with the constructed tetrahedral AB_4_ model (Figure 5F). The tomographic reconstruction based on STEM imaging—acquired over a tilt range of −60° to +60° with 1° increments—confirms that the crosslinked AB_4_‐type CMs retain a tetrahedron‐like 3D structure after drying (Figure 5G). Subtle structural differences between planar and 3D AB_4_‐type CMs are also manifested in their optical properties. Dark‐field scattering spectroscopy of individual assemblies shows that, although both geometries exhibit similar scattering colors (Figure S18, insets), the measured and simulated spectra consistently reveal that the scattering peak of the 3D AB_4_‐type CMs is red‐shifted by approximately 5 nm relative to that of their planar counterparts (Figure S18).

## 3 Conclusion

In conclusion, we have demonstrated a robust and mild ligand‐crosslinking strategy that markedly enhances the structural stability of both planar and 3D CMs by employing chemically optimized diazo compounds as crosslinkers. This approach offers several key advantages over existing stabilization methods for NP assemblies: (i) it preserves both the structural integrity and intrinsic physicochemical properties of the assemblies due to its gentle reaction conditions, (ii) it circumvents the need for tedious synthesis of bespoke reactive ligands and eliminates cumbersome post‐synthetic ligand modifications, and (iii) its inherently nonspecific reactivity affords broad applicability across diverse NP systems. This versatile and powerful stabilization platform establishes a critical foundation for CMs to hierarchically assemble into functional materials, mimic sophisticated molecular behaviors, and enable the construction of system‐level devices.

## Conflicts of Interest

The authors declare no conflicts of interest.

## Supporting information




**Supporting File**: advs74940‐sup‐0004‐SuppMat.docx

## Data Availability

The data that support the findings of this study are available from the corresponding author upon reasonable request.
