# Supplementary material for: Structurally Enhanced Colloidal Molecules via Carbene‐Mediated Covalent C─H Insertion Crosslinking
Source: Adv Sci (Weinh). 2026 Mar 23;13(31):e74940. doi: 10.1002/advs.74940 (PMC13588019; doi:10.1002/advs.74940)
Supplement: Supplementary file 1 [file ADVS-13-e74940-s001.docx]

**Structurally Enhanced Colloidal Molecules via Carbene-Mediated Covalent C−H Insertion Crosslinking**

Liwei Dai^a#^, Chongyang Yao^a#^, Xiancheng Zhang^a^, Weijia Kong^a^, Mingxing Liu^a^, Huibin He^a^*, Yutao Sang^a^*, and Zhihong Nie^a^*

^a^*State Key Laboratory of Molecular Engineering of Polymers, Department of Macromolecular Science, Fudan University, Shanghai 200438, P. R. China*

**Experimental section**

**1. Chemicals.**

Ethylene glycol (99%), pyridine (99%), dichloromethane (DCM, 99.9%), phenylacetyl chloride (99%), *p*-toluenesulfonyl azide (TsN_3_, 98%), 1,4-phenylenediacetic acid diethyl ester (98%), and 1,8-diazabicyclo-[5.4.0]-7-undecene (DBU, 98%) were acquired from Adamas. Ethyl acetate (≥99.5%), petroleum ether (AR), and glycerol (99%) were obtained from Greagent. 4-cyano-4-(phenylcarbonothioylthio)pentanoic acid (CPPA), anisole, azo-bis(isobutyronitrile) (AIBN), n-butylamine, sodium citrate, ascorbic acid (AA), and (11-mercaptoundecyl)-*N*,*N*,*N*-trimethylammonium bromide (TMA) were purchased from Sigma-Aldrich. Dioxane, tetrahydrofuran (THF), trifluoroacetic acid (TFA), Gold (III) chloride trihydrate (HAuCl_4_·3H_2_O, ≥99.9% trace metals basis), *N*,*N*-dimethylformamide (DMF), sodium chloride (NaCl), ethanol (anhydrous), methanol (anhydrous), and sodium hydroxide (NaOH) were purchased from Sinopharm Chemical Reagent Co., Ltd. Thiolated polyethylene glycol (PEG-SH) with various molecular weights (MW) were purchased from MeloPEG. AIBN was recrystallized twice from ethanol, and *tert*-butyl acrylate (*t*BA) was distilled under vacuum before use and stored at −18 ℃. All other chemicals were used as received unless otherwise specified.

**2. Synthesis of thiol-terminated poly (acrylic acid) (PAA-SH).**

The synthesis of PAA-SH was accomplished in three sequential steps.

**(1) Synthesis of P*t*BA-CTA.** The P*t*BA-CTA copolymer was synthesized via reversible addition-fragmentation chain-transfer (RAFT) polymerization. A mixture of *t*BA (60 mmol), CPPA (0.20 mmol), and AIBN (0.025 mmol) in dioxane (5 mL) was prepared in a 25 mL Schlenk flask. The mixture was degassed via three freeze-pump-thaw cycles. Polymerization was conducted at 70 °C for 24 hours under magnetic stirring and was terminated by rapid cooling in liquid nitrogen. The product was purified by dilution with THF, followed by precipitation in a methanol/water mixture (v/v = 1:1) three times. The resulting red precipitate was dried under vacuum at 50 °C overnight, yielding approximately 50% of P*t*BA-CTA.

**(2) End-group reduction to P*t*BA-SH.** To generate the thiol end-group, P*t*BA-CTA (2.5 g) was dissolved in THF (20 mL) under a N_2_ atmosphere. n-Butylamine (6.4 mmol) was then added, and the reaction proceeded at room temperature for 3 hours. The product (P*t*BA-SH) was purified by three cycles of precipitation in a methanol/water mixture (v/v = 1:1) and re-dissolution in THF, followed by drying under vacuum at 50 °C overnight. Gel permeation chromatography (GPC) determined the molecular weight (*M*_n_) of P*t*BA-SH to be 24.1 kg·mol⁻¹ with a dispersity (*Đ*) of 1.19, corresponding to a polymer structure of P*t*BA_188_-SH.

**(3) Hydrolysis to PAA-SH.** P*t*BA-SH (2.0 g) was dissolved in DCM (40 mL) under N_2_. TFA (10 mL) was added, and the hydrolysis was carried out at room temperature for 24 hours. During this process, the resulting PAA product gradually precipitated out of solution due to its poor solubility in DCM. Excess DCM was removed by rotary evaporation, and residual TFA was eliminated by three cycles of dissolution in methanol and precipitation in ethyl acetate. The final polymer, PAA_188_-SH, was obtained after drying under vacuum at 50 °C overnight, with an *M*_n_ of 13.5 kDa.

**3. Synthesis of spherical gold nanoparticles (Au NPs).**

Au NPs were synthesized according to a reported method with modifications.^1-2^ For the synthesis of 15 nm Au NPs, HAuCl_4_·3H_2_O aqueous solution (0.24 mL, 50 mg·mL⁻¹) was injected into 150 mL of boiling water under vigorous stirring. Subsequently, sodium citrate aqueous solution (4.5 mL, 10 mg·mL⁻¹) was quickly added. The mixture was refluxed for 30 minutes and then cooled to room temperature.

To obtain larger Au NPs, a seed-mediated growth approach was employed using the as-synthesized 15 nm Au NPs as seeds.^1-2^ In a typical procedure for 20 nm Au NPs, the seed solution was maintained at 90 °C. An additional 4.5 mL of sodium citrate solution (10 mg·mL⁻¹) and 0.24 mL of HAuCl_4_·3H_2_O solution (50 mg·mL⁻¹) were sequentially injected. The reaction mixture was heated to 100 °C under stirring, refluxed for 30 minutes, and then cooled to 90 °C. After repeating this growth cycle once, 20 nm Au NPs were obtained.

To synthesize 25 nm diameter Au NPs, a seed solution was prepared by adding 40 mL of 15 nm Au NPs and 80 mL of deionized water to a 250 mL three-neck round-bottom flask. The precursor solution was prepared by dissolving 0.4 mL of HAuCl₄·3H₂O (50 mg/mL) in 12.1 mL of deionized water. The reductant solution was prepared by mixing 1.25 mL of sodium citrate aqueous solution (1 wt%), 2.5 mL of AA aqueous solution (1 wt%), and 8.75 mL of deionized water. Using a two-channel syringe pump, 10 mL of the precursor solution and 10 mL of the reductant solution were simultaneously injected into the seed solution under vigorous stirring (800 rpm) at room temperature, at a flow rate of 10 mL/h. The solution was then heated and refluxed for 30 minutes before being allowed to cool to room temperature, yielding Au NPs with a diameter of 25 nm.

**4. Functionalization of Au NPs with PAA-SH ligands**

140 mL of the as-prepared Au NP solution was centrifuged to remove excess sodium citrate and then re-dispersed in 14 mL of DMF. Subsequently, 4 mL of a PAA-SH solution in DMF was added dropwise to the Au NP dispersion in a 40 mL glass vial under sonication. The mixture was sonicated for 30 minutes and then subjected to vortex mixing for 1000 minutes to ensure complete ligand exchange. The concentrations of Au NPs and PAA-SH employed for surface modification are provided in Table S1. The resulting PAA-functionalized Au NPs were purified through four cycles of centrifugation at room temperature and finally re-dispersed in 9 mL of deionized water for further use, unless otherwise specified.

**5. Functionalization of Au NPs with PEG-SH and TMA ligands**

160 mL of the as-prepared Au NP solution was centrifuged and then re-dispersed in 16 mL of deionized water. Next, 3 mL of a PEG-SH solution was added dropwise to the Au NP dispersion under stirring in a 40 mL glass vial. The mixture was stirred for an additional 10 hours to allow complete ligand binding, after which it was centrifuged to remove unbound PEG-SH. The resulting PEG-modified Au NPs were re-dispersed in 18 mL of deionized water. Subsequently, 1.8 mL of an aqueous TMA solution was introduced dropwise into the PEG-functionalized Au NP dispersion under continuous stirring. The reaction was allowed to proceed for 1 hour, followed by an 8-hour incubation period. The concentrations of Au NPs, PEG-SH, and TMA employed are listed in Table S1. Finally, the PEG/TMA grafted Au NPs were purified via four centrifugation cycles and re-dispersed in 9 mL of deionized water for future use, unless otherwise specified.

**6. Preparation of AB_x_-type colloidal molecules (CMs).**

AB_2_-type CMs were prepared using a dialysis-assisted self-assembly strategy. Briefly, an aqueous NaCl solution (0.1 M) was added to the individual NP-A and NP-B dispersions to obtain final NaCl concentrations of approximately 0.08–8 mM. The two NP dispersions were then combined in a 1.5 mL polypropylene centrifuge tube and subjected to sonication for 20 s. The resulting mixture was transferred into a dialysis membrane and dialyzed against deionized water for 24 hours to yield the CMs. Preparation of AB_4_-type CMs followed an identical procedure, except that NP-As and NP-Bs were replaced with NP-A′s and NP-B′s, respectively.

**7. Synthesis of** **ethyl phenyldiazoacetate (*1G* crosslinkers).**

A solution of 1,4-phenylenediacetic acid diethyl ester (2.5 g, 10 mmol) and TsN_3_ (5.91 g, 30 mmol) in DCM (40 mL) was stirred at room temperature. To this solution, DBU (4.56 g, 30 mmol) was added dropwise. After stirring for 12 hours, the resulting orange mixture was purified by silica gel column chromatography (ethyl acetate/petroleum ether = 1:30, v/v) to yield the desired product as an orange solid (2.23 g, 4.03 mmol, 74% yield).

**8. Synthesis of** **ethane-1,2-diyl bis(2-diazo-2-phenylacetate) (*2G* crosslinkers).**

The crosslinker *2G* was synthesized in two stages. Firstly, a solution of ethylene glycol (1.2 g) and pyridine (3.06 g) in DCM (120 mL) was prepared. Phenylacetyl chloride (6.56 g) was added dropwise to this solution at room temperature. After stirring for 12 hours, the mixture was purified by silica gel column chromatography (ethyl acetate/petroleum ether = 1:10, v/v) to afford the intermediate, 1,2-bis(phenylacetoxy) ethane (5.65 g, 98% yield). Subsequently, a solution of this intermediate (5.43 g) and TsN_3_ (7.9 g) in DCM (120 mL) was treated dropwise with DBU (6.08 g) at room temperature. The reaction mixture was stirred for 12 hours and then purified by silica gel column chromatography (ethyl acetate/petroleum ether = 1:50, v/v), yielding the final product *2G* as a yellow solid (5.98 g, 90% yield).

**9. Synthesis of** **propane-1,2,3-triyl tris(2-diazo-2-phenylacetate) (*3G* crosslinker).**

The crosslinker *3G* was synthesized in two stages. First, a solution of glycerol (0.92 g) and pyridine (3.08 g) in DCM (150 mL) was treated dropwise with phenylacetyl chloride (6.03 g) at room temperature. After stirring for 12 hours, the mixture was purified by silica gel column chromatography (ethyl acetate/petroleum ether = 1:10, v/v) to afford the intermediate, 1,2,3-tris(phenylacetoxy) propane (4.1 g, 92% yield). In the second stage, this intermediate (2 g) and TsN_3_ (2.91 g) were dissolved in DCM (60 mL), to which DBU (2.25 g) was added dropwise. Subsequently, additional portions of TsN_3_ (5.82 g) and DBU (4.5 g) were added dropwise in four equal batches at 12-hour intervals. The reaction was stirred for 72 hours and then purified via silica gel column chromatography (ethyl acetate/petroleum ether = 1:50, v/v), yielding *3G* as an orange oil (1.36 g, 58% yield).

**10. Non-specific crosslinking of CMs**

After centrifugation of 0.25 mL of an aqueous CM solution, the collected CMs were redispersed in DMF. A crosslinker (0–6 μmol) was then introduced into the CM dispersion. The mixture was irradiated with 254 nm UV light to induce non-specific C–H insertion between the crosslinker and the ligands on the CM surface. The crosslinked CMs were subsequently recovered by centrifugation and finally redispersed in an aqueous solution. It should be noted that all crosslinking experiments were performed under ambient conditions.

**11. COMSOL simulations.**

Optical simulations of the CMs were performed using COMSOL Multiphysics 6.1. The scattering-field equation in the “Electromagnetic Waves, Frequency Domain” module was employed, and a background electric field with wavelengths spanning 400–700 nm was applied to a spherical simulation domain enclosing the target nanostructures. The extinction cross-section ($\sigma_{extinction}$) was used to quantify the UV–vis absorption of the CMs, defined as:

$\sigma_{extinction}=\sigma_{absorption}+\sigma_{scattering}$	(1)

$\sigma_{scattering}=\frac{2Z_{0}}{E_{0}^{2}}∯ \vec{S_{p}}\cdot d\vec{A}$	(2)

$\sigma_{absorption}=\frac{2Z_{0}}{E_{0}^{2}}∰ Q_{e} d\tau$	(3)

where $Z_{0}$ is the vacuum impedance constant, $E_{0}$ is the amplitude of the background electric field, $\vec{S_{p}}$ is the Poynting vector, and $Q_{e}$ is the electromagnetic power-loss density. The scattering cross-section ($\sigma_{scattering}$) was calculated on a closed surface surrounding the CMs, whereas the absorption cross-section ($\sigma_{absorption}$) was obtained directly from its volume. To account for random orientations of CMs in solution, simulations were conducted under multiple incident-field propagation and polarization directions.

Perfectly matched layers (PMLs) and scattering boundary conditions were applied at the outer boundaries, and the external current density in the solvent domain was set to zero. The background refractive index was fixed at 1.33, corresponding to water. The diameters of the NP-As and NP-Bs were 20 nm and 15 nm, respectively, with interparticle spacing varied from 1.2 to 7.4 nm. A free tetrahedral mesh was used for the physical domain, while a swept mesh was applied to the PMLs. The maximum mesh sizes near and outside the nanostructure were set to 56.4 nm and 1.2 nm, respectively. The permittivity of Au was adopted from the experimental measurements^3^. All electric-field distributions were evaluated at the wavelengths corresponding to the plasmonic resonance peaks.

**12. Computational modeling of scattering spectra for 3D and planar AB_4_-type CMs.**

Finite-difference time-domain (FDTD) simulations were performed to investigate the optical response of the CMs. The complex permittivity of gold was adopted from the experimental data reported by Johnson and Christy^4^. The surrounding medium was defined as air with a refractive index of *n* = 1, and a silicon substrate (*n* = 1.45) was included in the model. A uniform mesh size of 1 nm was applied throughout the simulation region. A total-field scattered-field source was used to simulate extinction spectra in the wavelength range of 200–800 nm.

**13. Characterization Techniques.**

Transmission electron microscopy (TEM) and cryo-TEM images were acquired using a Hitachi HT7800 microscope and a JEOL JEM-Z300FSC microscope, respectively. High-angle annular dark-field scanning transmission electron microscopy (HAADF-STEM) images for 3D reconstruction were obtained with a Thermo Fisher Scientific Talos F200X G2 microscope. The hydrodynamic diameter and zeta potential of the NPs were measured at 20 °C using a Malvern Zetasizer-Nano 90. Scanning electron microscopy (SEM) images were obtained with a Zeiss Gemini SEM500 microscope. UV−vis absorption spectra were recorded with a PerkinElmer Lambda 1050+ two-beam spectrometer. Scattering spectra of CMs were collected using a Leica dark field microspectroscopy imaging system. Fourier-transform infrared (FTIR) spectra were acquired with a Thermo Fisher Nicolet 6700 spectrometer.

**Table S1.** Concentrations of the constituents used for the preparation of building blocks (i.e., NP-A, NP-A′, NP-B, and NP-B′).

**Figure S1.** Schematic illustration of the rational design of the diazo-based crosslinker. The chemical structures of (A) *1G*, (B) *2G*, and (C) *3G*.

**Figure S2.** ^1^H NMR spectrum of *1G* crosslinkers in CDCl_3_.

**Figure S3.** ^1^H NMR spectrum of *2G* crosslinkers in CDCl_3_.

**Figure S4.** ^1^H NMR spectrum of *3G* crosslinkers in CDCl_3_.

**Figure S5.** Synthetic route of *1G* crosslinkers.

**Figure S6.** Synthetic route of *2G* crosslinkers.

**Figure S7.** Synthetic route of *3G* crosslinkers.

**Figure S8.** (A) The molar extinction coefficients (*ε*) of the *1G*, *2G*, and *3G* crosslinkers. (B) Variation in the relative intensity of their characteristic absorption peaks as a function of UV irradiation time.

**Figure S9.** (A, C, E) TEM images and (B, D, F) corresponding size distribution histograms of Au NPs with different diameters.

**Figure S10.** Variation in the hydrodynamic diameter (*D*_h_) of PAA-grafted (left) and PEG/TMA-grafted (right) Au NPs versus pH.

**Figure S11.** Variation in the zeta potential of PAA-grafted (left) and PEG/TMA-grafted (right) Au NPs versus pH.

**Figure S12.** SEM images of crosslinked AB_2_-type CMs with different amounts of *3G*: (A) 0 μmol, (B) 0.5 μmol, (C) 1.5 μmol, (D) 3 μmol, and (E) 6 μmol. When low amounts of *3G* (0–1.5 μmol) were used, the crosslinked AB_2_-type CMs dissociated into free NP-A and AB structures under alkaline conditions (Figure S12A–C). In contrast, when the amount of *3G* reached or exceeded 3 μmol, the crosslinked AB_2_-type CMs maintained their original structure under the same conditions (Figure S12D, E).

**Figure S13.** (A–C) SEM images and (D) the statistical proportions of crosslinked AB_2_-type CMs under alkaline conditions.

**Figure S14.** SEM images of the uncrosslinked AB_2_-type CMs dispersed in a 10 mM NaCl solution.

**Figure S15.** (A) Cryo-TEM image of crosslinked AB_2_-type CMs and (B) corresponding statistical distributions of the interparticle spacing within the CMs.

**Figure S16.** Changes in electric field intensity near the bond region as a function of bond length.

**Figure S17.** (A) Schematic illustration (top), TEM images (middle), and STEM-HAADF images (bottom) of a uncrosslinked AB_4_-type CMs, captured at successive 10° rotations about the *Y*-axis. (B) 3D reconstruction of uncrosslinked AB_4_-type CMs (left), with multiple viewing angles of a single 3D model (right). The 3D model was generated primarily from TEM images acquired at 1° intervals over a *Y*-axis tilt range of ±60°.

**Figure S18.** Experimental and simulated scattering spectra of uncrosslinked (up) and crosslinked (bottom) AB_4_-type CMs.

**References**

1.	Yao, C.; He, H.; Kong, W.; Hu, L.; Shen, X.; Tao, J.; Sang, Y.; Nie, Z. Polymer-Driven Co-Assembly of Achiral and Chiral Nanoparticles into Plasmonic Nanoclusters with Quantitatively Modulated Optical Chirality. *Adv. Sci.* **2025,** *12*, e04850.

2.	He, H.; Shen, X.; Yao, C.; Tao, J.; Chen, W.; Nie, Z.; Wu, Y.; Dai, L.; Sang, Y. Hierarchically Responsive Alternating Nano-Copolymers with Tailored Interparticle Bonds. *Angew. Chem. Int. Ed.* **2024,** *63*, e202401828.

3.	Yakubovsky, D. I.; Arsenin, A. V.; Stebunov, Y. V.; Fedyanin, D. Y.; Volkov, V. S. Optical constants and structural properties of thin gold films. *Opt. Express* **2017,** *25*, 25574.

4.	Johnson, P. B.; Christy, R. W. Optical Constants of the Noble Metals. *Phys. Rev. B* **1972,** *6*, 4370-4379.
